# Treatment choices for fevers in children under-five years in a rural Ghanaian district

**DOI:** 10.1186/1475-2875-9-188

**Published:** 2010-06-28

**Authors:** Justice Nonvignon, Moses KS Aikins, Margaret A Chinbuah, Mercy Abbey, Margaret Gyapong, Bertha NA Garshong, Saviour Fia, John O Gyapong

**Affiliations:** 1 Department of Health Policy, Planning & Management, School of Public Health, College of Health Sciences, University of Ghana, PO Box LG 13, Legon, Ghana; 2 Dodowa Health Research Centre, PO Box DD 1, Dodowa, Ghana; 3 Research and Development Division, Ghana Health Service, PO Box 184, Accra, Ghana

## Abstract

**Background:**

Health care demand studies help to examine the behaviour of individuals and households during illnesses. Few of existing health care demand studies examine the choice of treatment services for childhood illnesses. Besides, in their analyses, many of the existing studies compare alternative treatment options to a single option, usually self-medication. This study aims at examining the factors that influence the choices that caregivers of children under-five years make regarding treatment of fevers due to malaria and pneumonia in a rural setting. The study also examines how the choice of alternative treatment options compare with each other.

**Methods:**

The study uses data from a 2006 household socio-economic survey and health and demographic surveillance covering caregivers of 529 children under-five years of age in the Dangme West District and applies a multinomial probit technique to model the choice of treatment services for fevers in under-fives in rural Ghana. Four health care options are considered: self-medication, over-the-counter providers, public providers and private providers.

**Results:**

The findings indicate that longer travel, waiting and treatment times encourage people to use self-medication and over-the-counter providers compared to public and private providers. Caregivers with health insurance coverage also use care from public providers compared to over-the-counter or private providers. Caregivers with higher incomes use public and private providers over self-medication while higher treatment charges and longer times at public facilities encourage caregivers to resort to private providers. Besides, caregivers of female under-fives use self-care while caregivers of male under-fives use public providers instead of self-care, implying gender disparity in the choice of treatment.

**Conclusions:**

The results of this study imply that efforts at curbing under-five mortality due to malaria and pneumonia need to take into account care-seeking behaviour of caregivers of under-fives as well as implementation of strategies.

## Background

Malaria and pneumonia are a major cause of under-five morbidity and mortality worldwide. Both diseases share many characteristics, including fever and signs of severe illness, such as inability to eat, convulsions and difficult breathing. According to the World Health Organization (WHO), malaria is prevalent in more than 100 countries worldwide and about 1.2 billion people representing 20% of the world's population are at high risk of malaria, with 49% of this population living in Africa [1]. In 2006, 91% of the world's 881,000 malaria deaths occurred in sub-Saharan Africa (SSA).

Malaria and pneumonia are both leading causes of deaths in children under-five years of age in Africa. Estimates show that 85% of the malaria deaths in Africa occurred in children under-five years of age and 150 million episodes of pneumonia occur annually in under-fives in developing countries including Africa, representing more than 95% of all new cases worldwide [1,2].

In Ghana, malaria and acute respiratory infections (principally pneumonia) are the leading causes of fever in children. Malaria is responsible for the majority of childhood admissions and 22% of childhood deaths while pneumonia is also responsible for about 22% of hospital admissions in tertiary health facilities [3,4]. Current control strategies for both malaria and pneumonia include early diagnosis and prompt treatment, particularly among those at risk of death and severe complications. Until recently when the Ministry of Health introduced community interventions, the official interventions for both illnesses were largely geared towards the formal health sector, even though many people (especially in rural areas) have little or no physical access to health facilities - i.e. live beyond 30 minutes travel time from facilities[5]. The result is that many caregivers of children seek treatment from outside the home (usually non-hospitals sources) [6] or self-treat.

Alternative treatment options for childhood illnesses in Ghana can, broadly, be categorized into: self-care or self-medication, care from over-the-counter (OTC) providers, public providers (public health centres, clinics, and hospitals), private providers (private health centres, clinics, hospitals). Self-care is an indigenous form of treatment. Self-care refers to care at the home and comes in the form of caregiver diagnosis, tepid sponging (i.e. washing the sick child's body with warm water to reduce temperature) and/or treatment using self-made herbal remedies or left-over prescriptions and/or doing nothing. Non-formal providers exist in rural Ghana. These include traditional healers, spiritualists, and drug peddlers. Chemical sellers are sometimes put into this group because, even though chemical stores are supposed to be manned by people specifically trained to diagnose illnesses and give first aid treatment, the reality is that most of such stores are operated by untrained people [7].

Studies on treatment of fevers and other illnesses in children submit that the level of education of the caregiver's partner and other members of a household are as important as the education of the caregiver in influencing the choice of treatment [8,9]. Besides, households who watched television at least once in a week and those who have used a health facility at least once in a year are more likely to use modern health care compared to traditional or no care [8].

In developing countries, the quality of health care services influences the demand for these services [10,11]. Besides, individuals in small, literate households are more likely to choose private health facilities instead of public facilities [12].l The demand for care at clinics tends to reduce with larger household sizes [13]. Physical access to health facility is also an important factor influencing the demand for health care services i.e. inaccessibility to care services tend to reduce the demand for care [14]. Further, higher household incomes encourage the use of the desired formal health care services [15]. Travel and treatment times are associated with reduction in utilization of formal health services [7,14].

Studies in Ghana and Tanzania have stressed the role of prices in influencing health care utilization [10,15-17]. These studies argue that when the cost of health care services rises, the demand for such services will fall. Regarding sex, females tend to seek the desired external care compared to males [7,10,18]. Also, households with more female children tend to use self-care compared to care from hospitals while those with more male adults use care from drugstores over self-care [18]. However, lower income and longer distance to facility tend to reduce female utilization of services while better education, worse health status and lower costs tend to increase male utilization of health services [19].

The objective of this paper is to empirically examine the factors which influence the demand for treatment services for fevers in children under-five years of age in a rural Ghanaian district. Such analysis is important in shaping understanding of the utilization of health care services for treatment of childhood illnesses. It also helps to inform policies regarding childhood illnesses in the country.

## Methods

### Study area

The Dangme West District is a rural district in the Greater Accra Region comprising 376 communities and has an estimated population of 109,459 (from 2009 Health and Demographic Surveillance data collected by the Dodowa Health Research Centre) with a land size of about 1,700 km sq. Approximately 13% of the population is below five years of age. It lies in the coastal savannah and is bounded on the North by the Akwapim Ranges; to the South by the gulf of Guinea, to the East by the River Volta and the Dangme East district and to the West by the Ga East District and Tema Municipality. It is flat relatively and at sea level with isolated hills. The district is divided into seven area councils and four administrative sub-districts. These administrative sub-districts correspond to the four traditional areas of the district i.e. the Shai, Prampram, Ningo and Osudoku traditional areas. They are also related to the district assembly division of the district into area councils.

### Type of study and data source

The study employs quantitative analysis. The data for the study come from two separate surveys- a baseline household survey on health-seeking behaviour conducted as part of a study on home management of fevers and a Demographic Surveillance System in the Dangme West District- both undertaken by the Dodowa Health Research Centre.

### Sample size

During the baseline survey, caregivers whose children under-five years of age had fever in the fourteen-day period that preceded the survey were interviewed and information was collected on the signs and symptoms of the illness, facility attended and cost of treatment. Demographic information on caregiver, household income and household coverage of health insurance were collected during demographic surveillance. This study uses a sample of 529 children, about whom data was available.

### Estimation technique

The estimation technique that this study uses follows the specification by Nonvignon and Aglobitse [18]. This study seeks to model a caregiver's selection of a health care provider, which is conditional on a child being ill. This is a discrete choice, and because there can be several choice alternatives, a multinomial response model is more appropriate.

The multinomial response model used here is motivated using a random utility framework. In this framework, the assumption is that an individual chooses a given health care option only if the utility the individual derives from the chosen option is greater than the utility that can be derived from an alternative option. Suppose that the utility that the *ith *individual derives from the *jth *alternative, *j = 1,...J*, is given as(1)

Where, the row vector *Z*_*i *_contains the observed independent variables for the *ith *decision maker. Associated with *Z*_*i *_are the vectors of regression coefficients *α*_*j*_. The *ξ*_*i*_,...,*ξ*_*i*,*J *_are error terms that capture the random components of the model. The decision maker chooses the alternative k if *U*_*ik *_≥ *U*_*im *_for *m *≠ *k*.

Supposing individual *i *selects alternative k, and taking the difference between *U*_*ik *_and the *J-1 *others, the following is arrived at:(2)

Where *j*' = *j *if *j *<*k *and *j*' = *j *- 1 if *j *>*k *so that *j*' = 1,...,*j *- 1.

The multinomial probit (MNP) model assumes that ∈_*i *_= (∈_*i*1_,...,∈_*i,J*-1_) follow a multivariate normal distribution. The probability that individual *i *chooses outcome *k *is given as(3)

Where *λ*_*ij' *_= *Z*_i_*γ*_*j'*_.

The above equation is solved to give the *K*- point quadrature formula(4)

Where, *w*_*k *_and *x*_*k *_are the weights and roots respectively of the Laguerre polynomial of order *K*.

Even though equation (2) can result in specification of both multinomial logit (MNL) and MNP models, the latter was chosen because of an inherent property of MNL known as independence of irrelevant alternatives (IIA), which is arrived at if the error terms in equations (1) and (2) are assumed to be independent and random. The IIA property in MNL implies that the probability of choosing one health care option does not depend on presence (or absence) of other options, which is not always true. An earlier study submits that in Ghana, the choice of one health care alternative sometimes depends on the presence (or absence) of another [18].

Four health care options are considered in this study. These are self-medication, care from over-the-counter providers, care from public providers, and care from private providers. Unlike previous health care demand studies, this study estimates four separate MNP models, varying the base category (with which other alternatives are compared) in each case. This is because, even though many previous studies use self-care as comparison option, it is important to show how the choice of a given alternative varies compared to each of the other alternatives.

### Variables and *a-priori *expectations

The dependent variable in this study is the choice of a fever treatment service. Four options fall under this category. These are self-medication, over-the-counter providers, public providers and private providers. Self-medication includes sponging, usage of drugs at home and doing nothing. Over-the-counter providers include drug peddlers, chemical sellers, herbalists, and prayer camps. Public providers include government-funded clinics and hospitals while private providers include private clinics as well as mission clinics.

The independent variables used in the study are age, sex of the sick child, sex of the one who contributes most money for treatment, household's monthly income and health insurance status, education of caregiver, treatment cost and time (i.e. travel, waiting and treatment times as one variable).

The variable "education" represents education of caregiver measured by the number of years of schooling and was treated as continuous variable. For all sex variables, dummies were imposed, with values of 0 for females and 1 for males. Also, dummy was imposed on health insurance status with values of "0" for uninsured and "1" for insured households. Income, age and treatment cost were treated as continuous variables. Health insurance status and income represent the entire household while age represents the age of the caregiver.

*A priori*, a positive relationship is expected between age of caregiver and the probability of choosing a facility as older people are more likely to seek care from external formal providers compared to younger people [13].

A number of studies have shown that households tend to self-medicate or use over-the-counter treatment sources if treatment cost is high at health care facilities [10,13,15]. This study relies on the same expectation. Also, households headed by females tend to use formal care compared to those headed by males while households with more male children often use formal external health care services compared with those with more female children [18]. This study expects the same results.

Further, households with higher incomes are expected to use public or private facilities relative to self-medication or over-the-counter care [7]. The probability of choosing formal health care facilities is expected to increase as with higher education [10]. This is because more educated people better understand the disadvantages of self-medication and would want to avoid using such sources, other things being equal. This study also expects that households with health insurance coverage will opt for formal health service providers since they will not have to pay anything at the point of services. Also, travel, waiting and treatment times are expected to be negatively related to the probability of seeking care from public and private providers, that is, higher times to and at these facilities will encourage caregivers to resort to self-medication.

## Results

Seventy-five percent of fever cases reported in the study were first managed using home-made remedies (e.g. sponging and use of left-over drugs) and services of over-the-counter providers. Table 1 presents the summary statistics of selected independent variables used in the estimation of the MNP model. The table indicates that 27 percent of the households surveyed were covered by a health insurance scheme. The current percentage might be higher because the survey took place not long after the introduction of the National Health Insurance Scheme. Also, the mean travel, waiting and treatment time is 1 hour 43 minutes.. This can be attributed to the small number of health facilities in the district; people have to travel longer and spend time waiting to be treated. Table 1 also shows that caregivers of children under-five years in the district have a mean age of 34 years and average years of schooling of 4.5 years.

**Table 1:** Summary statistics of selected independent variables

Variable	Observation	Mean	No. (%)
Age	526	34 (yrs)	
Education	529	4.5 (yrs)	
*Monthly Income	513	127.10 (GH¢)	
*Treatment cost	404	3.90 (GH¢)	
Time	523	1.43 (hrs)	
Sex of child (males)	298		274 (52)
HI status	529		144 (27)

* The exchange rate between the Ghana Cedi (GH¢) and United States Dollar in 2006 was US$1: GH¢0.92

The results of the multinomial probit model of treatment choice for fevers are presented in Tables 2, 3, 4 and 5. Four MNP models were estimated, in each case alternating the comparison group.

The results of the estimation as presented in Table 2 shows that caregivers of female under-fives use self-medication (or self-care) while caregivers of male under-fives use public providers compared to self-medication in the choice of treatment services for fevers in rural Ghana. Notwithstanding, the evidence for this conclusion is not strong enough since the marginal effect was 2 percent. Further evidence is required. Apart from this, the study finds that in the Dangme West District, the age of a caregiver and the sex of the one who contributes most to the health care of under-fives do not significantly influence the choice of fever treatment services.

Of the socio-economic variables used in the estimation, monthly household income and health insurance status of households were statistically significant; the results show that caregivers with higher incomes use public or private providers compared to self-medication (Tables 2) while households with health insurance coverage use care from public providers compared to over-the-counter providers (Tables 3 and 4). The study also indicates that households with health insurance coverage use care from public providers compared to private providers. Surprising as it might seem, the level of education of caregivers does not turn out to significantly influence fever treatment choices in the Dangme West district.

The findings (as shown in Tables 4 and 5) also indicate that longer travel, waiting and treatment times at public and private facilities tend to encourage caregivers to use self-medication or over-the-counter providers and this is significant at 1 percent. However, Table 5 shows that higher times at public facilities encourage caregivers to use private facilities. Tables 2 and 3 also show that higher treatment charges by public and private providers encourage caregivers to use these facilities and this is quite surprising and does not meet expectations. Also, Tables 4 and 5 indicate that higher treatment charges by public health service providers encourage caregivers to resort to the use of private providers (and this is significant at 1 percent).

**Table 2:** Multinomial probit regression results with self-medication as comparison group

Variables	Option		
	
	OTC	Public	Private
log of age	0.041	0.869	0.717
	(0.551)	(0.751)	(0.899)
sex of child	0.396	0.645*	0.218
	(0.283)	(0.385)	(0.467)
sex of payer	0.074	0.258	0.074
	(0.316)	(0.419)	(0.497)
log of income	0.172***	0.220***	0.235**
	(0.073)	(0.084)	(0.106)
Education	0.000	0.016	-0.014
	(0.037)	(0.046)	(0.056)
HI status	-0.230	0.530	-0.515
	(0.339)	(0.460)	(0.594)
log of trt. cost	0.184*	0.375**	1.957***
	(0.127)	(0.179)	(0.324)
Time	0.008	-0.041***	-0.026***
	(0.005)	(0.006)	(0.007)
Constant	-2.882	-7.943**	-25.040***
	(2.533)	(3.451)	(5.096)

Log pseudo likelihood = -214.24853

Number of observations = 403

Wald chi square (24) = 180.09

Probability level of significance based on Wild chi square = 0.000

Notes: ***, **, * denotes significant at 1%, 5%, and 10% respectively.

Values are coefficients, with robust standard errors in parentheses

**Table 3:** Multinomial probit regression results with over-the-counter (OTC) as comparison group

Variables	Option		
	
	Self-med.	Public	Private
log of age	0.041	0.911	0.759
	(0.551	(0.634)	(0.799)
sex of child	-0.396	0.250	-0.178
	(0.283)	(0.317)	(0.411)
sex of payer	-0.074	0.183	-0.000
	(0.316)	(0.349)	(0.440)
log of income	-0.172***	0.048	0.063
	(0.073)	(0.079)	(0.099)
Education	-0.000	0.015	-0.014
	(0.037)	(0.038)	(0.049)
HI status	0.229	0.760**	-0.285
	(0.339)	(0.387)	(0.540)
log of trt. cost	-0.184	0.191	1.773***
	(0.127)	(0.160)	(0.310)
time	-0.008*	-0.049***	-0.034***
	(0.005)	(0.005)	(0.006)
Constant	2.882	-5.061*	-22.158***
	(2.533)	(3.034)	(4.761)

Log pseudo likelihood = -214.24853

Number of observations = 403

Wald chi square (24) = 180.09

Probability level of significance based on Wild chi square = 0.000

**Table 4:** Multinomial probit regression results with public providers as comparison group

Variables	Option		
	
	Self-med.	OTC	Private
log of age	-0.869	-0.911	-0.152
	(0.751)	(0.634)	(0.739)
sex of child	-0.645*	-0.250	-0.427
	(0.385)	(0.317)	(0.395)
sex of payer	-0.258	-0.183	-0.184
	(0.419)	(0.349)	(0.411)
log of income	-0.220***	-0.048	0.015
	(0.084)	(0.079)	(0.099)
education	-0.016	-0.015	-0.029
	(0.046)	(0.037)	(0.045)
HI status	-0.530	-0.760**	-1.045**
	(0.460)	(0.387)	(0.509)
log of trt. Cost	-0.375**	-0.191	1.582***
	(0.179)	(0.160)	(0.296)
time	0.041***	0.049***	0.014**
	(0.006)	(0.005)	(0.007)
Constant	7.943**	5.062*	-17.097***
	(3.451)	(3.034)	(4.435)

Log pseudo likelihood = -214.24853

Number of observations = 403

Wald chi square (24) = 180.09

Probability level of significance based on Wild chi square = 0.000

**Table 5:** Multinomial probit regression results with private provider as comparison group

Variables	Option		
	
	Self-med.	OTC	Private
log of age	-0.717	-0.759	0.152
	(0.898)	(0.799)	(0.739)
sex of child	-0.218	0.178	0.427
	(0.467)	(0.411)	(0.395)
sex of payer	-0.074	0.000	0.184
	(0.497)	(0.440)	(0.411)
log of income	-0.235**	-0.063	-0.015
	(0.106)	(0.099)	(0.099)
Education	0.014	0.014	0.029
	(0.056)	(0.049)	(0.045)
HI status	0.515	0.285	1.045**
	(0.594)	(0.540)	(0.509)
log of trt. Cost	-1.957***	-1.773***	-1.582***
	(0.324)	(0.310)	(0.296)
time	0.026***	0.034***	-0.014**
	(0.007)	(0.006)	(0.007)
Constant	25.040***	22.158***	17.097***
	(5.096)	(4.761)	(4.435)

Log pseudo likelihood = -214.24853

Number of observations = 403

Wald chi square (24) = 180.09

Probability level of significance based on Wild chi square = 0.000

## Discussion

More than half of fever cases reported in the study were first managed using home-made remedies and services of over-the-counter providers. This confirms the findings that poly-pharmacy is a common practice in Ghana [6,7]. Previous studies report similar findings in other countries [8]. This finding brings to light the importance of home management programmes where communities will be allowed to manage fever cases through the services of community volunteers trained by the health sector to diagnose and administer appropriate treatment while referring more complicated cases to health facilities. Such strategies do not only promote community participation, but also eliminates the problem of physical inaccessibility to health care services (i.e. longer travel times) while coping with the problem of inadequate staff at health facilities.

The findings of the study also confirm the well-known fact that household income is an important factor that influences the choice of health care services as caregivers with higher incomes tend to use formal health facilities compared to self-medication. The Dangme West District is rural with predominantly farming and fishing activities and, perhaps, the long term and best way to improve incomes of households will be by implementing economic policies that favour small scale farmers and fishermen in the country. Currently, small-scale farmers are faced with numerous challenges, including the use of primitive methods and lack of markets for their farm produce. Small scale fishermen, especially along the coast, get low catch because larger trawlers fish close to the coast, driving the fish away. Any policy aimed at favouring these farmers and fishermen will need to address these issues. That will help them increase productivity and find good markets for their produce. Not only will their access to health facilities be enhanced, but also their standards of living will improve, reducing vulnerability to illnesses.

The finding that caregivers of female under-fives use self-medication while caregivers of male under-fives use public providers compared to self-medication confirms the finding by previous studies that there is gender disparity in health service utilization in Ghana [7,10,18] despite efforts by the Government of Ghana and other stakeholders to reduce (if not totally eliminate) gender inequity in the patronage of social services such as education and health. Thus, moving strongly towards achieving Millennium Development Goal number four (MDG4) of reducing child mortality requires that the role of child's gender in influencing caregiver's choice of treatment services need not be overlooked even though the size of the marginal effects is small.

Further, the result that households with health insurance coverage use care from public providers compared to over-the-counter providers implies that even in its early years, the National Health Insurance Scheme (NHIS) seems on course to achieving its aim of encouraging the use of formal health services during illnesses. Coverage of the NHIS needs expansion. However, the result that households with health insurance coverage use care from public providers compared to private providers might have a link with unsubstantiated reports that some accredited private facilities do not treat NHIS card-bearing patients the same way as patients who pay cash. Such reports need to be investigated by the NHIS and actions taken to solve the issue. Further, other bottlenecks in implementation of the scheme such as the delays in issuance of identity cards for those who register with the scheme need to be eliminated to encourage households who have not registered with the scheme to do so.

Again, the result that longer travel, waiting and treatment times at public and private facilities encourage caregivers to use self-medication and over-the-counter sources confirms expectations. In Ghana as in other developing countries, travel and waiting times are high especially in rural areas (as indicated on Table 1 which presents the mean time to be 1 hour 43 minutes) and this situation is better in private facilities than in public ones. There are fewer health facilities and fewer health personnel to attend to the large number of patients- the doctor/population ratio in Ghana is 1:13,683 [20]. Thus, ways of encouraging caregivers to seek formal treatment for their children will be to either to increase health personnel or move health services closer to the people. The latter seems more feasible given the fact that it takes a long time to train health personnel, especially doctors. Home management programmes could help in this regard.

Further, the results show that higher treatment charges by public health facilities or high charges by private facilities tend to encourage the use of private facilities. Surprising as this might be, in reality, people perceive higher charges by private providers to mean higher quality.

## Conclusions

The study sought to examine the factors that influence the choices that caregivers of children under-five years of age in Dangme West district make regarding treatment of fevers. The study finds that longer travel, waiting and treatment times at public and private facilities encourage people to resort to the use of self-medication or over-the-counter providers. Also, caregivers with health insurance coverage use care from public providers compared to over-the-counter or private providers. The study also finds that caregivers with higher incomes use public and private providers over self-medication while higher treatment charges as well as longer times at public facilities encourage caregivers to resort to private providers. Finally, the findings indicate that caregivers of female under-fives use self-care while caregivers of male under-fives use public providers to self-medication.

It is important to state that this study is useful in indicating that efforts at curbing under-five mortality due to malaria and pneumonia need to take into account care-seeking behaviour of caregivers of under-fives as well as implementation of strategies. However, care must be taken in relating the interpretations of the findings of this study to the situation in urban areas. This is because the factors influencing treatment choices in rural areas might differ from those influencing choices in urban areas given that urban areas have more facilities than rural areas. Also, the weak performance of relevant variables such as treatment cost and education of caregiver forms a basis for further research.

Future research can improve upon this work by using data from both rural and urban areas, and by including quality of service as a variable in the estimation of the demand for care.

## Competing interests

The authors declare that they have no competing interests.

## Authors' contributions

JN conceived the study, conducted the analysis and interpretation and drafted the manuscript. MKSA reviewed the conception, analysis and the draft manuscript. MAC, MA, BNAG and MG designed the data collection tools and were instrumental in the acquisition of data. SF contributed to the analysis of the data. JOG was instrumental in the acquisition of funding, general supervision and reviewed the concept and draft manuscript. All authors read and approved the final manuscript.
